# Tubulin Cytoskeleton Organization in Cells of Determinate Nodules in *Vigna radiata*, *Vigna unguiculata,* and *Lotus corniculatus*

**DOI:** 10.3390/plants14192986

**Published:** 2025-09-26

**Authors:** Anna B. Kitaeva, Pyotr G. Kusakin, Artemii P. Gorshkov, Anna V. Tsyganova, Viktor E. Tsyganov

**Affiliations:** Laboratory of Molecular and Cell Biology, All-Russia Research Institute for Agricultural Microbiology, Saint Petersburg 196608, Russia; akitaeva@arriam.ru (A.B.K.); pyotr.kusakin@arriam.ru (P.G.K.); a.gorshkov@arriam.ru (A.P.G.); avtsyganova@arriam.ru (A.V.T.)

**Keywords:** legume–rhizobial symbiosis, microtubules, symbiosome, bacteroid, determinate nodules, *Vigna* sp., *Lotus corniculatus*

## Abstract

Tubulin cytoskeleton rearrangements play an important role in the cell differentiation of symbiotic nodules in legumes. However, the organization of the tubulin cytoskeleton has been investigated only for four legume species forming determinate nodules (with limited nodule meristem activity). In this study, microtubule organization was studied in three species (*Vigna radiata*, *V*. *unguiculata*, and *Lotus corniculatus*) with determinate nodules using confocal laser scanning microscopy and quantitative analyses. Histological organization in young nodules of *V. radiata* and *V. unguiculata* resembled the recently reported zonation in young nodules of *Glycine max*. In addition, bacteroids in nodules of these species were significantly enlarged compared to free-living bacteria. Organization of endoplasmic and cortical microtubules in young infected cells and uninfected cells and that of cortical microtubules in nitrogen-fixing cells demonstrated general patterns for determinate nodules, whereas endoplasmic microtubules in nitrogen-fixing cells showed species-specific patterns. Thus, the presence of both general and species-specific patterns of tubulin cytoskeleton organization was confirmed in determinate nodules.

## 1. Introduction

Mungbean (*Vigna radiata* (L.) R. Wilczek) is an important legume crop of East and Southeast Asia [1], while cowpea (*Vigna unguiculata* (L.) Walp.) is widespread in Africa and South America [2]. Both of these pulse crops are drought-resistant and susceptible to waterlogging [3,4,5,6]. *Lotus corniculatus* L. is a forage legume of Mediterranean origin, which is able to adapt to different environments, such as water deficits, nutritional deficiencies, and low temperatures [7]. All three species form determinate nodules. Such nodules are typical for legumes from warm climates (like soybean (*Glycine max* (L.) Merr.) and common bean (*Phaseolus vulgaris* L.)). In determinate nodules, cell division is activated in cells of the middle or outer cortex [8,9]. Rhizobia colonize these cells via transcellular infection threads and are released from unwalled infection droplets [10]. Released bacteria continue to divide and form multibacteroid symbiosomes containing rod-shaped, nonswollen (i.e., similar in shape and size to free-living bacteria) bacteroids [11]. However, it should be noted that individual bacteroids in determinate nodules of different species can be significantly increased in size and pleiomorphic in shape [12,13,14]. Meristematic activity in determinate nodules is transient, and the growth of a nodule involves cell enlargement. As a result, mature determinate nodules are round-shaped and lack clear histological zonation [15]. The central part of mature determinate nodules, consisting of infected and uninfected cells, is surrounded by parenchyma, endodermis, and cortex peripheral tissues [16,17]. In contrast to determinate nodules, in indeterminate nodules cell divisions are induced in the pericycle and inner cortical layers [18]. The meristem in such nodules originates from the root middle cortex [19] and functions for a long-term period, resulting in the formation of histological zonation (meristem, infection zone, nitrogen fixation zone, and senescence zone) [15]. Bacteroids in these nodules undergo significant differentiation and lose the ability to multiply [20]. Indeterminate nodules are typical of legumes growing in temperate latitudes (for example, pea (*Pisum sativum* L.) and alfalfa (*Medicago sativa* L.)).

Development of determinate nodules is accompanied with intensive cytoskeleton rearrangements of both actin microfilaments and microtubules [21]. In general, in plant cells, the tubulin cytoskeleton consists of cortical and endoplasmic arrays of microtubules. Cortical microtubules regulate the direction of cell growth, form preprophase bands, and are involved in transport and tethering of organelles, while endoplasmic microtubules form mitotic spindles and phragmoplasts during mitosis [22,23]. At early stages of nodule development, dynamics of microtubules and forming patterns direct the growth of the infection thread and guide nucleus movement in a root hair [24,25]. Microtubules are also associated with the formation of pre-infection threads, which are specialized transcellular cytoplasmic bridges [18]. In a mature nodule, the organization of the tubulin cytoskeleton was studied in different legume species forming determinate [12,26] and indeterminate [27,28,29,30] nodules. The comprehensive analyses revealed general and species-specific patterns of tubulin cytoskeleton organization. In the meristem cells of nodules of all analyzed species, cortical microtubules formed an irregular pattern, located at different angles, and endoplasmic microtubules linked the nucleus with the periphery of the cell. In young infected cells in nodules of all studied species, endoplasmic microtubules were associated with infection threads and infection droplets, determining the direction of their growth. In nitrogen-fixing cells, endoplasmic microtubules formed a network among symbiosomes. Moreover, for different species of legumes forming indeterminate nodules, irregular, regular, or intermediate patterns of their organization were characteristic, while in determinate nodules only an irregular pattern was apparent. The revealed differences are probably associated with pronounced differences in the morphology of bacteroids observed between various species of legumes with indeterminate nodules. In uninfected cells, cortical microtubules formed an irregular pattern in nodules of *G. max* and *Glycine soja* Siebold & Zucc., whereas the other species had a regular pattern with microtubules oriented transversely to the longitudinal axis of the cell. In indeterminate nodules in infected cells, cortical microtubules formed an irregular pattern, while in mature cells of determinate nodules, they formed a regular one. However, the number of species studied is still very limited and the involvement of new legume species is necessary to confirm the universality of the identified general patterns, as well as the identification of new species-specific ones.

In this work, bacteroid morphology, nodule histological organization, and organization of the tubulin cytoskeleton in different types of nodule cells of *V. radiata*, *V. unguiculata*, and *L. corniculatus* forming determinate nodules were studied.

## 2. Results

### 2.1. Histological Organization of Nodules

**Figure 1:**
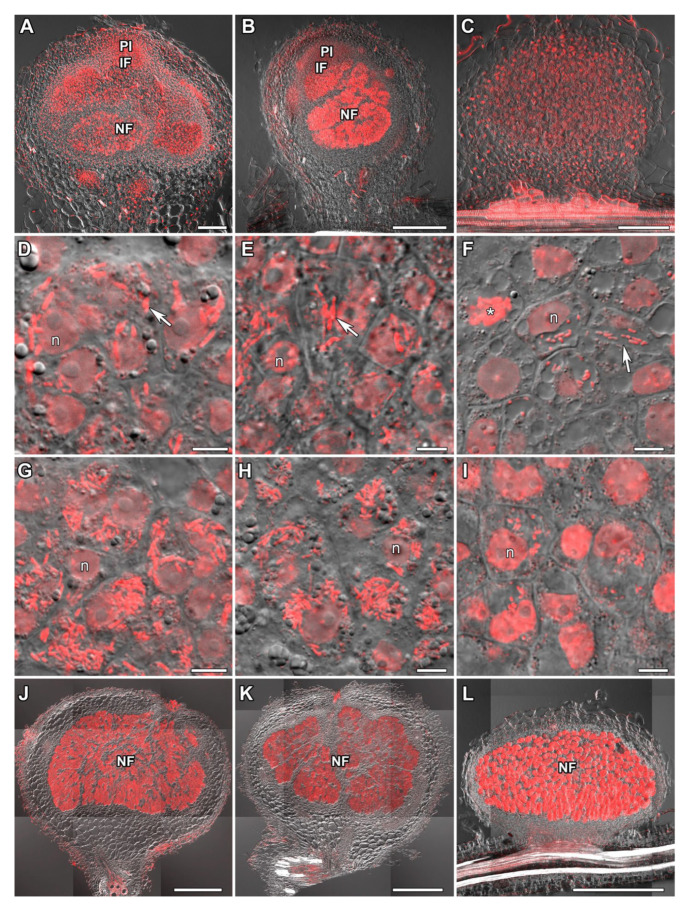
Histological structure of nodules. (**A**,**D**,**G**,**J**) *Vigna radiata*, (**B**,**E**,**H**,**K**) *Vigna unguiculata*, and (**C**,**F**,**I**,**L**) *Lotus corniculatus*. Laser scanning confocal microscopy. Merged images of differential interference contrast and red channel (DNA staining with propidium iodide). (**A**–**I**) Nodules 11 days after inoculation. (**J**–**L**) Nodules 28 days after inoculation. (**A**–**C**,**J**–**L**) General view of a nodule. In nodules of *V. radiata* and *V. unguiculata*, a “pre-infection zone” (**D**,**E**) and “infection zone” (**G**,**H**) were identified, but not in *L. corniculatus* nodules (**F**,**I**; see text for details). PI, pre-infection zone; IF, infection zone; NF, nitrogen fixation zone; n, nucleus; asterisk indicates mitotic figure; arrows indicate infection threads. Bars are 100 µm (**A**–**C**), 5 µm (**D**–**I**), and 500 µm (**J**–**L**).

The studied species formed determinate nodules, the histological organization of which differed slightly (Figure 1). In 11-day-old nodules of *V. radiata* and *V. unguiculata*, infection threads and small cells containing a few released bacteria were found in the distal part of the nodules (Figure 1D,E; presumably the pre-infection zone [31]). In the more proximal part, the number of released bacteria increased (Figure 1G,H; presumably the infection zone [31]). In the central part of nodules, young infected cells increased in size and were filled with numerous bacteria (Figure 1A,B; presumably the nitrogen fixation zone [31]). In contrast, in 11-day-old nodules of *L. corniculatus*, the designation of such zones has been troublesome (Figure 1C,F,I). The studied species differed in the arrangement of uninfected cells: in nodules of *V. radiata* and *V. unguiculata*, they formed groups and rows, while in *L. corniculatus* they only formed groups (Figure 1A–C).

In 16- and 28-day-old nodules of all studied species, all infected cells increased significantly in size, and cells located in the basal part were elongated (Figure 1J–L). Uninfected cells maintained their distribution (Figure 1J–L).

### 2.2. Morphology of Bacteria and Bacteroids

**Figure 2:**
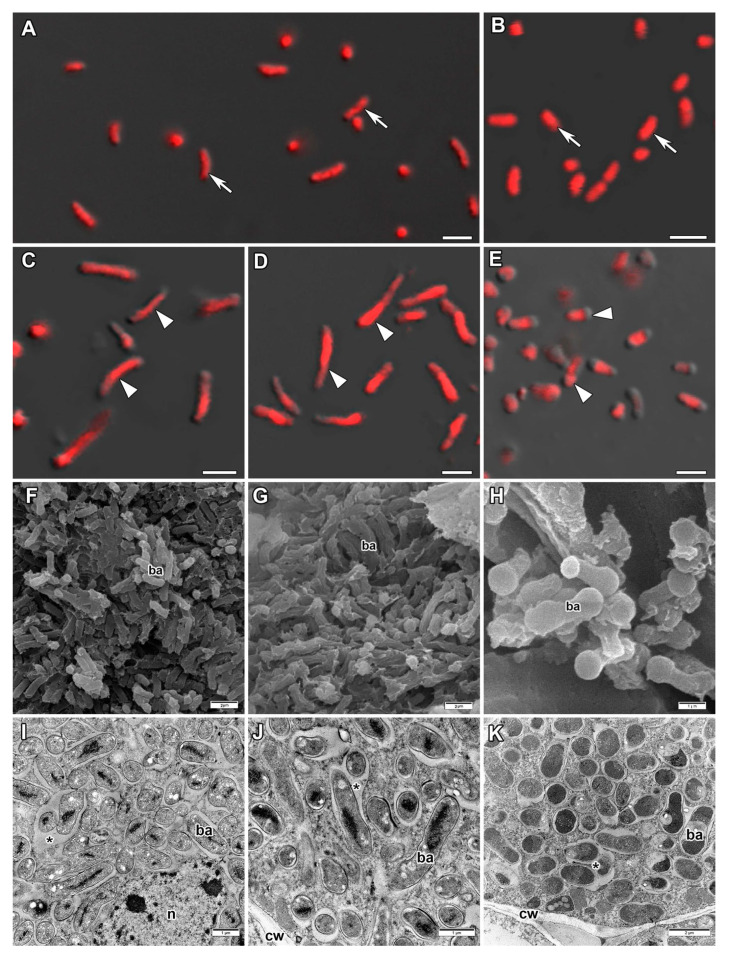
Morphology of bacteria and bacteroids. (**A**) *Bradyrhizobium ottawaense* RCAM0503 strain used for inoculation of *Vigna radiata* and *Vigna unguiculata*. (**B**) *Mesorhizobium loti* RCAM1804 strain used for inoculation of *Lotus corniculatus*. (**C**,**F**,**I**) Bacteroids from *V. radiata* nodules. (**D**,**G**,**J**) Bacteroids from *V. unguiculata* nodules. (**E**,**H**,**K**) Bacteroids from *L. corniculatus* nodules. (**A**–**E**) Laser scanning confocal microscopy. Merged images of differential interference contrast and red channel (DNA staining with propidium iodide). (**F**–**H**) Scanning electron microscopy. (**I**–**K**) Transmission electron microscopy. ba, bacteroid; cw, cell wall; n, nucleus; arrows indicate bacteria; arrowheads indicate bacteroids; asterisks indicate multibacteroid symbiosomes. Bars are 2 µm (**A**–**G**,**K**) and 1 µm (**H**–**J**).

**Figure 3:**
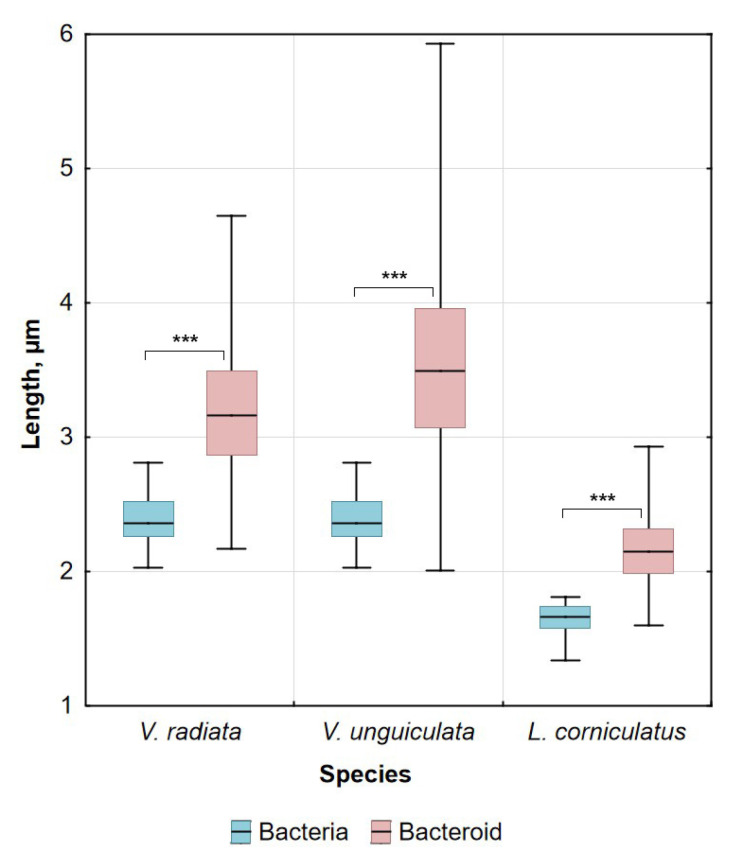
Length of free-living bacteria and bacteroids in nitrogen-fixing cells of *Vigna radiata*, *Vigna unguiculata*, and *Lotus corniculatus*. Pairwise comparisons were conducted using Mann–Whitney’s test, *** *p* < 0.01; n = 99 for bacteria and n = 202 for bacteroids.

Free-living bacteria of *Bradyrhizobium ottawaense* RCAM0503 were characterized by a rod-shape (Figure 2A) and had a length of about 2.5 µm (Figure 3). Most parts of *V. radiata* bacteroids had a length of about 2.8–3.5 µm, while bacteroids of *V. unguiculata* were longer and were about 3–4 µm in length (Figure 3). The shape of *V. radiata* (Figure 2C,F) and *V. unguiculata* (Figure 2D,G) bacteroids ranged from rod-shaped to elongated when studied using confocal and electron scanning microscopy. In ultrastructural analyses of the nodules of both *Vigna* species, most symbiosomes contained one bacteroid each, and multibacteroid symbiosomes were uncommon (Figure 2I,J). Bacteroids had a translucent matrix and a centrally located nucleoid of increased electron density (Figure 2I,J).

Bacteria of *Mesorhizobium loti* RCAM1804 were characterized by being rod-shaped (Figure 2B) and the length was about 1.7 µm (Figure 3). The length of *L. corniculatus* bacteroids was longer and was about 2–2.3 µm (Figure 3). In nodules of *L. corniculatus*, the bacteroids were rod- or dumbbell-shaped (Figure 2H). When studying the ultrastructure of these nodules, the uneven density of the bacteroid matrix with an increase in density at the tips was noticeable (Figure 2K), which may explain the dumbbell shape of bacteroids in electron scanning microscopy. Most parts of symbiosomes contained a single bacteroid, while multibacteroid symbiosomes were also present (Figure 2K).

### 2.3. Microtubule Organization in Young Infected Cells

**Figure 4:**
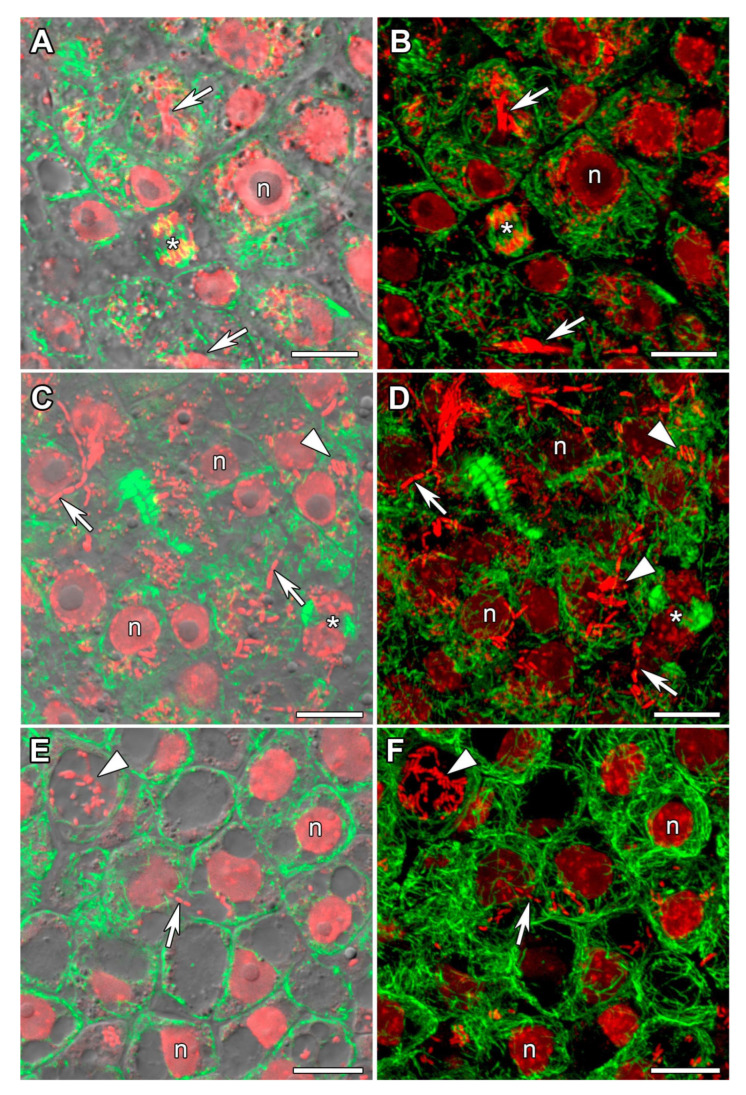
Microtubule organization in young infected cells. (**A**,**B**) *Vigna radiata*, (**C**,**D**) *Vigna unguiculata*, (**E**,**F**) and *Lotus corniculatus*. Confocal laser scanning microscopy of 35 µm longitudinal vibratome sections. Immunolocalization of tubulin (microtubules), green channel; DNA staining with propidium iodide (nuclei and bacteria), red channel. (**A**,**C**,**E**) Merged images of a single optical section of differential interference contrast, green, and red channels. (**B**,**D**,**F**) Maximum intensity projections of 10 (**B**) and 15 (**D**,**F**) optical sections in green and red channels. n, nucleus; asterisks indicate mitotic figures; arrowheads indicate infection droplets; arrows indicate infection threads. Bars are 10 µm.

In all studied species, the organization of the tubulin cytoskeleton in young infected cells was similar (Figure 4). Infection threads, infection droplets, and released bacteria were observed in young infected cells. In these cells, cortical microtubules criss-crossed and formed a dense network. Endoplasmic microtubules formed a net around the nucleus; they were observed among released bacteria. In cells involved in mitosis, microtubules formed spindles and phragmoplasts (Figure 4A–D).

### 2.4. Microtubule Organization in Uninfected Cells

**Figure 5:**
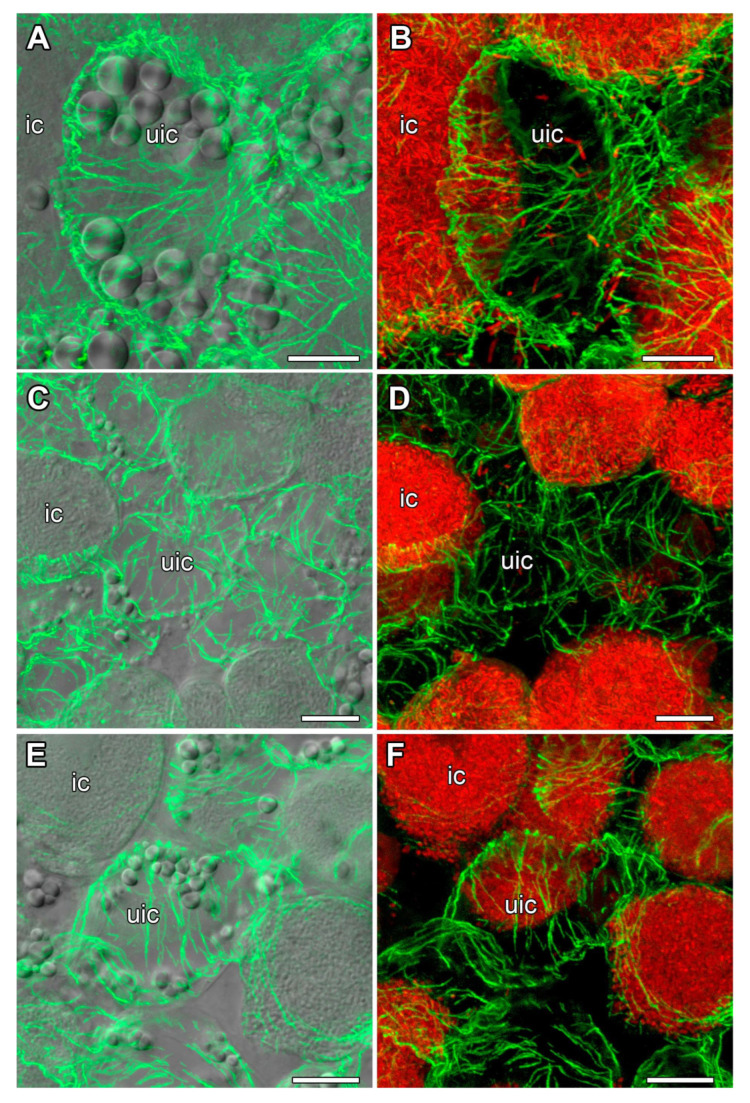
Cortical microtubule organization in uninfected cells. (**A**,**B**) *Vigna radiata*, (**C**,**D**) *Vigna unguiculata*, and (**E**,**F**) *Lotus corniculatus*. Confocal laser scanning microscopy of 50 µm (**A**–**D**) and 35 µm (**E**,**F**) longitudinal vibratome sections. Immunolocalization of tubulin (microtubules), green channel; DNA staining with propidium iodide (nuclei and bacteria), red channel. (**A**,**C**,**E**) Merged images of a single optical section of differential interference contrast and maximum intensity projection of 40 (**B**), 45 (**D**), and 22 (**F**) optical sections in green channel. (**B**,**D**,**F**) Maximum intensity projections of green and red channels. ic, infected cell; uic, uninfected cell. Bars are 10 µm.

**Figure 6:**
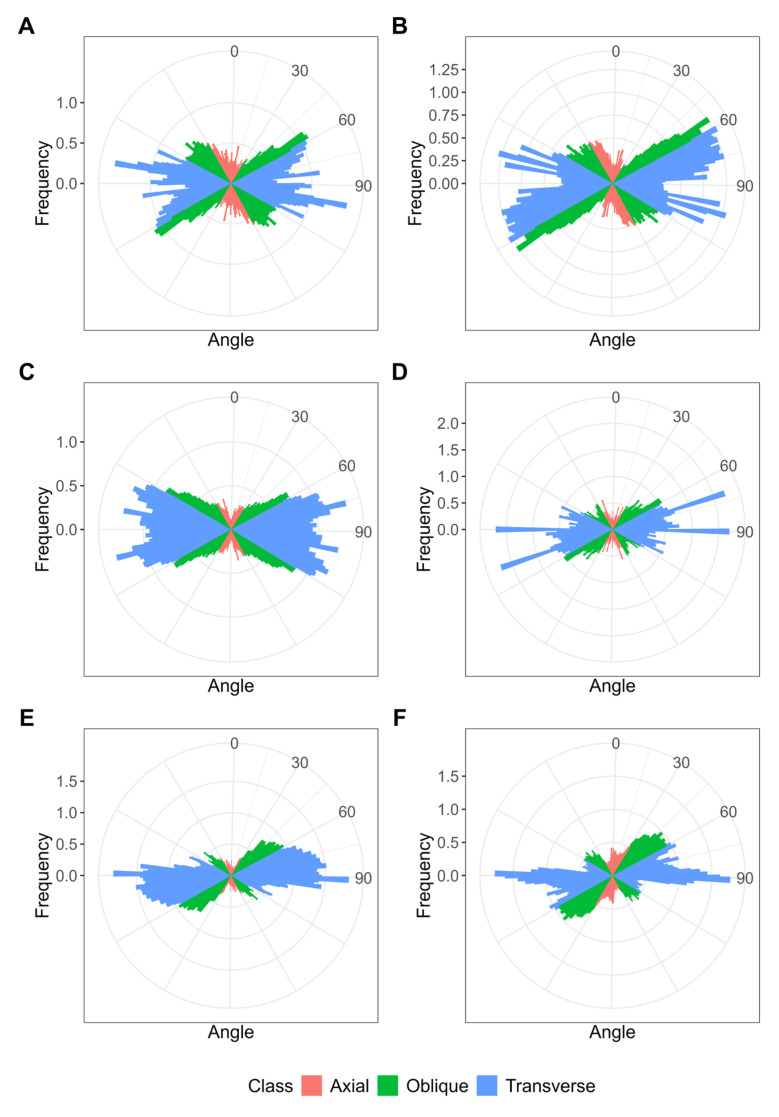
Quantitative analysis of cortical microtubule orientation in uninfected (**A**,**C**,**E**) and infected (**B**,**D**,**F**) cells of *Vigna radiata* (**A**,**B**), *Vigna unguiculata* (**C**,**D**), and *Lotus corniculatus* (**E**,**F**) nodules. Color indicates the class of angles of the microtubules relative to the longitudinal axis of the cell: red, axial (0–30°, 150–180°); green, oblique (30–60°, 120–150°); blue, transverse (60–120°). Normalized and averaged histograms for 10–15 cells are presented.

In all three species, cortical microtubules formed bundles relatively parallel to each other and transverse to the longitudinal axis of a cell. Thus, they displayed a regular pattern (Figure 5). Visual observations were confirmed by the quantitative analysis (Figure 6A,C,E). In uninfected cells of all studied species, a small portion of endoplasmic microtubules was associated with amyloplasts and was connected with the cell periphery (Figure S1).

### 2.5. Organization of Cortical Microtubules in Nitrogen-Fixing Cells

**Figure 7:**
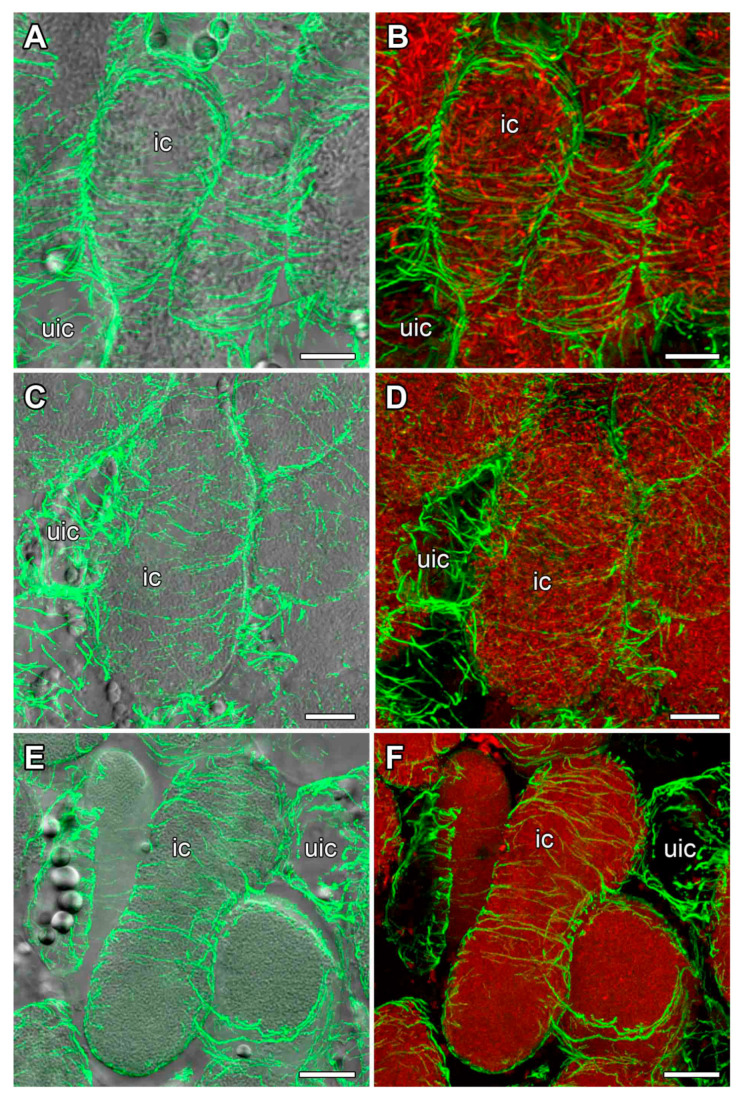
Cortical microtubule organization in nitrogen-fixing cells. (**A**,**B**) *Vigna radiata*, (**C**,**D**) *Vigna unguiculata*, and (**E**,**F**) *Lotus corniculatus*. Confocal laser scanning microscopy of 50 µm (**A**–**D**) and 35 µm (**E**,**F**) longitudinal vibratome sections. Immunolocalization of tubulin (microtubules), green channel; DNA staining with propidium iodide (nuclei and bacteria), red channel. (**A**,**C**,**E**) Merged images of a single optical section of differential interference contrast and maximum intensity projection of 40 (**B**,**F**) and 45 (**D**) optical sections in green channel. (**B**,**D**,**F**) Maximum intensity projections of green and red channels. ic, infected cell; uic, uninfected cell. Bars are 10 µm.

In nitrogen-fixing cells, cortical microtubules formed parallel bundles transverse to the longitudinal axis, showing a regular pattern (Figure 7). Quantitative analysis showed that the cortical microtubules were mostly transverse (Figure 6B,D,F).

### 2.6. Organization of Endoplasmic Microtubules in Nitrogen-Fixing Cells

**Figure 8:**
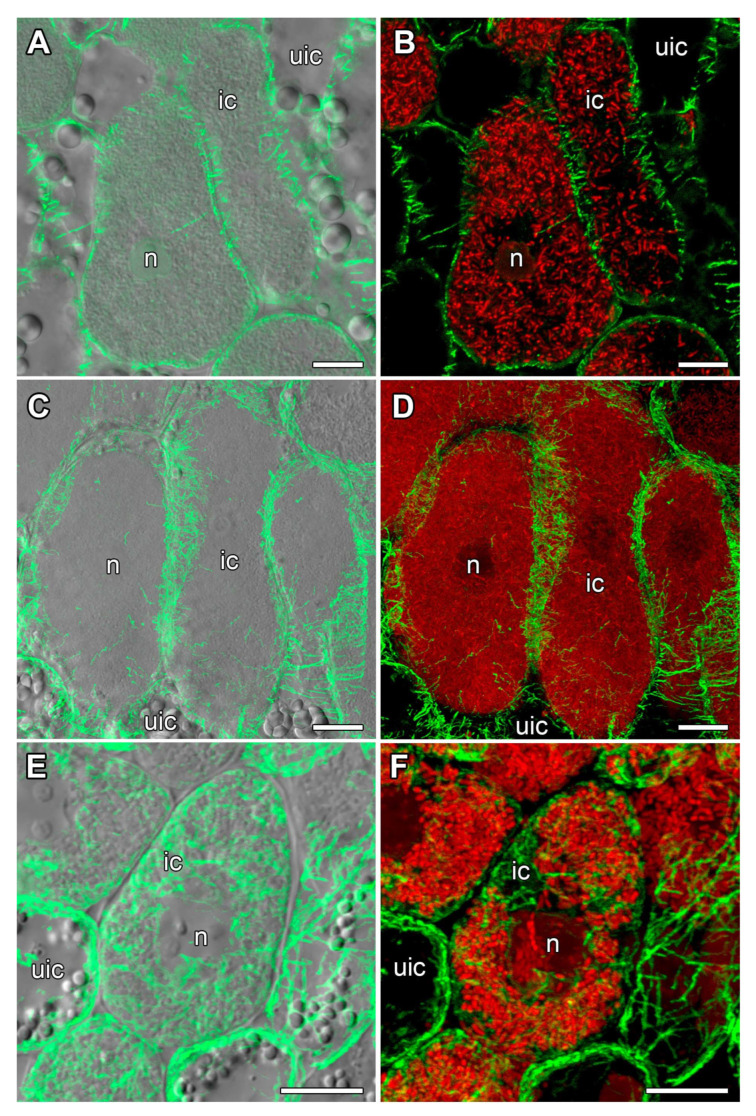
Endoplasmic microtubule organization in nitrogen-fixing cells. (**A**,**B**) *Vigna radiata*, (**C**,**D**) *Vigna unguiculata*, and (**E**,**F**) *Lotus corniculatus*. Confocal laser scanning microscopy of 50 µm (**A**–**D**) and 35 µm (**E**,**F**) longitudinal vibratome sections. Immunolocalization of tubulin (microtubules), green channel; DNA staining with propidium iodide (nuclei and bacteria), red channel. (**A**,**C**,**E**) Merged images of a single optical section of differential interference contrast and maximum intensity projection of 25 (**B**) and 30 (**D**,**F**) optical sections in green channel. (**B**,**D**,**F**) Maximum intensity projections of green and red channels. n, nucleus; ic, infected cell; uic, uninfected cell. Bars are 10 µm.

In nitrogen-fixing cells of *V. radiata* and *V. unguiculata*, rare wavy endoplasmic microtubules were observed among symbiosomes (Figure 8A–D). In contrast, in nitrogen-fixing cells of *L. corniculatus* nodules, highly branched endoplasmic microtubules were revealed (Figure 8E,F).

## 3. Discussion

The study of tubulin cytoskeleton organization in determinate nodules has only recently been conducted for four legume species [12]. As a result, common patterns of microtubule organization were identified in the nodules of *P. vulgaris*, *Lotus japonicus* (Regel) K. Larsen, *G. max*, and *G. soja*. At the same time, the organization of cortical microtubules in uninfected nodule cells of both *Glycine* species differed markedly from that in *P. vulgaris* and *L. japonicus*, as well as in indeterminate nodules [12,27,28,29,30]. In this study, we investigated the organization of the tubulin cytoskeleton in determinate nodules of three another legume species belonging to the Phaseoleae tribe (*V. radiata* and *V. unguiculata*) and the Loteae tribe (*L. corniculatus*) [32]. We expected to detect regular microtubule patterns in infected and uninfected cells, although identification of an irregular pattern in uninfected cells was also possible, as well as an irregular endoplasmic microtubule pattern in infected cells.

In this study, in the distal part of 11-day-old nodules of *V. radiata* and *V. unguiculata*, we observed infection threads and small cells containing a few released bacteria (Figure 1A,B,D,E). Young enlarged infected cells filled with numerous bacteria occupied the central part of the nodule (Figure 1A,B,G,H). Previously, in 11-day-old nodules of *V. radiata*, infection threads and infected cells were described in the central zone [14]. The infection threads and small cells with a small number of bacteria in the distal part of *V. radiata* and *V. unguiculata* nodules reported here resemble the pre-infection and infection zones recently identified in young *G. max* nodules [31]. The central part of the nodule, as in our study and in a study by Newcomb and McIntyre [14], corresponds to the nitrogen fixation zone in soybean nodules. It is noteworthy that histological zonation was not distinguished in determinate nodules, but Tu et al. [31] showed that it is present in young soybean nodules, and its disappearance is associated with the activation of the enzyme GRETCHEN HAGEN3, which leads to the formation of auxin conjugates and its inactivation, triggering cell differentiation. Uninfected cells in the nodules of both *Vigna* species formed groups and rows, dividing the nodule into lobes (Figure 1A,B,J,K). This organization is characteristic of determinate nodules of *V. radiata* [14] and soybean [33], which may be associated with the transport of ureides during nitrogen fixation [34].

It is noteworthy that no structures that could be associated with pre-infection and infection zones of *G. max* were observed in *L. corniculatus* nodules (Figure 1C,L). Also, uninfected cells did not form rows. No differences in histological organization of *L. corniculatus* nodules with previous investigations [35] were revealed.

Nodules of determinate type are characterized by nonswollen bacteroids, which do not change significantly in size and form compared with free-living bacteria [11,36]. In the current study, most part of bacteroids in *V*. *radiata* nodules were 25% longer than free-living bacteria. However, bacteroids two times longer than free-living bacteria were also observed. Elongated and several pleomorphic bacteroids were previously observed in nodules of *V*. *radiata* [14]. In our research, *V*. *radiata* plants formed nodules with symbiosomes containing mostly only one bacteroid. However, it was previously shown that when inoculated with the *Rhizobium* sp. 440 strain, the symbiosomes of *V. radiata* nodules contained up to 10 bacteroids [14]. Bacteroids in nodules of *V*. *unguiculata* were longer comparing to *V*. *radiata* bacteroids, with an average length of 3.5 µm, and some reached up to 6 µm. It is interesting to note that in previous studies, bacteroids did not exceed the length of free-living bacteria upon inoculation of *V*. *unguiculata* plants with *Sinorhizobium* sp. strain NGR234 [37]. In this study, symbiosomes of *V*. *unguiculata* nodules contained only one bacteroid. A similar pattern was observed earlier when *V. unguiculata* was inoculated with *Rhizobium* sp. strain 32H1 [38]. In the current study, bacteroids in nodules of *L. corniculatus* were longer compared with free-living bacteria and were characterized by a dumbbell-like shape. It has previously been shown that when *L. corniculatus* were inoculated with *Rhizobium* sp., bacteroids were 50% longer compared with free-living bacteria and 10 or more bacteroids were enclosed with one symbiosome membrane [35].

In young infected cells of 11-day-old nodules from all three studied species, cortical microtubules were oriented in a criss-cross pattern and endoplasmic microtubules formed a network around the nuclei and were also located among juvenile bacteroids (Figure 4), as previously described for all studied legume species [12,27,29,30]. The identified phragmoplasts and spindles in the nodules of *V. radiata* and *V. unguiculata* indicated mitotic activity in cells from the presumed pre-infection and infection zones (Figure 4A–D).

In uninfected cells of 16-day-old nodules from all studied species, cortical microtubules formed a regular pattern, i.e., they were arranged parallel to each other and perpendicular to the longitudinal axis of the cell (Figure 5 and Figure 6). This pattern was characteristic of all previously described species forming both determinate and indeterminate nodules [12,27,28,29,30], with the exception of soybean [12,26]. In this study, short bundles of endoplasmic microtubules associated with starch grains were identified (Figure S1). Previously, endoplasmic microtubules were not detected in uninfected cells (Table 1 in [12]). However, it should be noted that their identification is difficult due to the vacuole occupying a significant part of the cell.

In infected cells of 16-day-old nodules from all three studied species, cortical microtubules formed a regular pattern (Figure 7). Previously, such a pattern had been described for mature infected cells of four legume species that formed determinate nodules [12,26]. Such a regular pattern of cortical microtubule organization is characteristic of anisotropic cell growth [22].

Endoplasmic microtubules in infected cells of 16-day-old nodules from *V. radiata* and *V. unguiculata* were difficult to detect; only rare wavy bundles were occasionally observed (Figure 8A–D). At the same time, a clearly distinguishable network of endoplasmic microtubules was detected in the nodules of *L. corniculatus* (Figure 8E,F). Nevertheless, in all species studied, the pattern of endoplasmic microtubules can be classified as irregular. Previously, such a pattern was shown for infected nodule cells of *P. vulgaris*, *L. japonicus*, *G. max*, and *G. soja*, although the density of the microtubule network differed between species [12].

In contrast to indeterminate nodules, symbiosomes of determinate nodules contain several bacteroids. It might be assumed that the pattern of endoplasmic microtubules in nitrogen-fixing cells of determinate nodules is associated with the size and shape of symbiosomes.

## 4. Materials and Methods

### 4.1. Plant Material and Bacterial Strains

The commercial seeds of *Vigna radiata* (L.) R. Wilczek var. *radiata* cv. ‘Pobeda 104’ and *Vigna unguiculata* (L.) Walp. ssp. *unguiculata* cv. ‘Dachnitsa’ were used. Seeds of *Lotus corniculatus* L. accession K-39329 from the Collection of Federal Research Center N. I. Vavilov All-Russian Institute of Plant Genetic Resources (VIR) were kindly provided by Dr. Margarita Vishnyakova.

Seeds were sterilized in concentrated sulfuric acid for 1 min, followed by washing with sterile water 10 times and germination at 28 °C in a Petri dish with wet filter paper. Seedlings were inoculated with 1 ml of water suspension of the corresponding rhizobial strain containing 10^7^–10^8^ cells per seed. All the strains used in experiments were taken from the Russian Collection of Agricultural Microorganisms (All-Russia Research Institute for Agricultural Microbiology). Both *Vigna* species were inoculated with *Bradyrhizobium ottawaense* RCAM0503 (NCBI RefSeq assembly: GCF_052180175.1). This strain was collected in Uzbekistan from *V. unguiculata* nodules. *L. corniculatus* was inoculated with *Mesorhizobium loti* RCAM1804.

Plants of both *Vigna* species were grown in sterile vermiculite wetted with nitrogen-free nutrient solution [39] in a JIUPO growth chamber (Fujian Jiupo Biotechnology Co., Fuzhou, China) under controlled conditions: day/night, 16/8; temperature, 28 °C day/24 °C night; humidity, 75%; illumination, 280 µmol photons m^−2^ s^−1^. *L*. *corniculatus* plants were grown in sterile quartz sand wetted with nitrogen-free nutrient solution [40] in an MLR-352H growth chamber (Sanyo Electric Co., Moriguchi, Japan) with controlled conditions: day/night, 16/8; temperature, 21 °C; humidity, 75%; illumination, 280 µmol photons m^−2^ s^−1^. The nodules were harvested on the 11th, 16th, and 28th days after the inoculation.

### 4.2. Microscopy

#### 4.2.1. Electron Microscopy

For electron microscopy, both transmission and scanning, 10–15 nodules from ten 28-day-old plants were collected for each variant. The nodules were transferred directly into a drop of 2.5% (*w*/*v*) glutaraldehyde diluted in 10 mM phosphate buffer (Sigma-Aldrich, St. Louis, MO, USA), containing 2.7 mM potassium chloride and 137 mM sodium chloride at pH 7.4. For better penetration of the fixative, the cortex was cut off on one side of each nodule. The samples were then transferred to plastic tubes and placed in a vacuum (4 times for 20 min with a 5 min break) to remove air from the intercellular space. After vacuum infiltration, the floating nodules were removed and the fixative was replaced with a fresh solution.

For transmission electron microscopy, the nodules were fixed overnight at 4 °C and then the samples were washed in 10 mM phosphate buffer (4 times for 10 min) and post-fixed in a 1% aqueous solution of osmium tetroxide in 10 mM phosphate buffer for 1 h (the fixative was prepared from a 2% solution (ChemMed, Moscow, Russia) by dilution). The nodules were then dehydrated in a series of solutions with increasing ethanol concentrations, with the final step being 100% acetone (Table S1). The dehydrated samples were gradually infiltrated with solutions of EMbed-812 epoxy resin (EMS, Hatfield, PA, USA) of increasing concentration mixed with 90% acetone (Table S1). All these procedures were performed in an EM TP tissue processor (Leica Microsystems, Vienna, Austria). The following medium-hardness EMbed-812 epoxy resin recipe was used for infiltration and embedding: 48% EMbed-812, 25% DDSA, and 27% NMA. DMP-30 was added at a concentration of 1% of the resin at the final polymerization stage. The samples were transferred for embedding into small 0.3 mL plastic containers with fresh resin, which were polymerized in an IN55 incubator (Memmert, Schwabach, Germany) at 60 °C for 48 h.

Ultrathin sections (90–100 nm thick) were cut using a Leica EM UC7 ultramicrotome (Leica Microsystems) with a diamond knife (Diatome, Nidau, Switzerland). Samples were put on the copper grids coated with formvar/carbon films (Electron Microscopy Sciences, Hatfield, PA, USA). The sections were contrasted with a 2% aqueous solution of uranyl acetate for 30 min and then with lead citrate for 1 min in an automatic contrast system for ultrathin sections of EM AC20 (Leica Microsystems) at 21 °C.

The samples were examined and photographed in a transmission electron microscope JEM-1400 (JEOL Corp., Tokyo, Japan) equipped with a Veleta CCD camera (Olympus-SIS, Münster, Germany) at 80 kV.

For scanning electron microscopy, the nodules were dehydrated with a graded ethanol series and dried with a critical point dryer, Leica EM CPD300 (Leica Microsystems). Then specimens were mounted on stubs, coated with 12 nm gold with the high-vacuum sputter coater Leica EM SCD500 (Leica Microsystems), and observed in a Jeol JSM 6390LA scanning electron microscope (JEOL) at 20 kV.

#### 4.2.2. Immunolocalization and Laser Scanning Confocal Microscopy

For histological organization analyses, 11-, 16-, and 28-day-old nodules were used, while 11- and 16-day-old nodules were used for tubulin cytoskeleton visualization. Immunolocalization of tubulin microtubules and staining of nuclei and bacteria were performed as described previously with some modifications [27]. For each species, the composition of fixative solution and the procedure for air pumping out were developed (Table S2). Nodule longitudinal sections were made using a microtome with a vibrating blade HM650V (Microm, Walldorf, Germany). For precise staining with propidium iodide, sections of nodules were incubated in RNAse A solution (Thermo Fisher Scientific, Waltham, MA, USA) in a dilution of 1:200 during 30 min at 28 °C. Sections were mounted in ProLong Diamond antifade reagent (Thermo Fisher Scientific). Microtubule pattern analyses in nodule cells were performed using an LSM 780 laser scanning confocal microscope and ZEN 2012 software (Zeiss, Oberkochen, Germany). AlexaFluor 488 was excited at 488 nm, and fluorescence emitted between 499 and 543 nm was collected; propidium iodide was excited at 561, and emitted fluorescence between 606 and 677 nm was collected.

### 4.3. Visualization of Free-Living Bacteria and Bacteroids

For visualization of free-living bacteria and bacteroids, a previously developed technique was used [29]. To analyze the difference in size between bacteria and bacteroids, the length of 99 bacteria and 202 bacteroids from 28-day-old nodules were measured. The Shapiro–Wilk test was used for normality (*p* < 0.01). Pairwise comparisons were conducted using Mann–Whitney’s test (*p* < 0.01).

### 4.4. Quantitative Analysis

Quantitative analysis of tubulin distribution patterns was performed as previously described [30] with some modifications. Extraction of whole cells and the cortical tubulin cytoskeleton from 3D confocal images was performed using a custom script for ImageJ’s (v. 1.53t) [41] built-in Python: regions of interest (ROIs) corresponding to cell shape or cortical microtubules were determined on several optical slices, which were then interpolated to the whole stack. To analyze microtubule orientation, images were converted to maximum intensity projections and thresholded, the longitudinal axis was determined for each cell, and the microtubule orientations were obtained using a Python script for ImageJ and the MicroFilament Analyzer software [42]. From 10 to 15 cell images were used for analyses for each variant.

## 5. Conclusions

In this study, cytoskeleton organization was analyzed in cells of determinate nodules of three more legume species. First of all, it is interesting to note that histological analysis of young nodules revealed cell organization in nodules of *V. radiata* and *V. unguiculata* resembling the recently identified zonation in young nodules of soybean. The study of the morphology of bacteroids in the nodules of the studied species showed that there was an increase in the size of bacteroids in both *Vigna* species compared to free-living bacteria. As we expected, the patterns of cortical microtubule organization in infected nodule cells were similar to those described for *P. vulgaris*, *L. japonicus*, *G. soja*, and *G. max*. and those in uninfected cells were comparable with those in nodules of *P. vulgaris* and *L. japonicus*. A developed network of endoplasmic microtubules was detected only in infected nodule cells of *L. corniculatus*, but not in *Vigna* species, while earlier such a network was detected in infected cells of all studied species forming determinate nodules. Thus, this study confirmed the presence of both general and species-specific patterns of tubulin cytoskeleton organization in determinate nodules.

## Data Availability

The data presented in this study are available in the article and supplementary material.

## Supplementary Materials

The following supporting information can be downloaded at https://www.mdpi.com/article/10.3390/plants14192986/s1: Figure S1: Endoplasmic microtubule organization in uninfected cells; Table S1: Protocol for sample preparation; Table S2: Composition of fixative solutions.
