# Supplementary material for: Tubulin Cytoskeleton Organization in Cells of Determinate Nodules in Vigna radiata, Vigna unguiculata, and Lotus corniculatus
Source: Plants (Basel). 2025 Sep 26;14(19):2986. doi: 10.3390/plants14192986 (PMC12525943; doi:10.3390/plants14192986)
Supplement: Supplementary file 1 [file plants-14-02986-s001.zip › plants-3836019-supplementary.pdf]

Electronic Supplementary Material

Tubulin Cytoskeleton Organization in Cells of Determinate Nodules in *Vigna radiata*, *Vigna unguiculata*, and *Lotus corniculatus*

Anna B. Kitaeva, Pyotr G. Kusakin, Artemii P. Gorshkov, Anna V. Tsyganova, and Viktor E. Tsyganov*

Laboratory of Molecular and Cell Biology, All-Russia Research Institute for Agricultural Microbiology, Saint Petersburg, 196608, Russia; akitaeva@arriam.ru (A.B.K.); pyotr.kusakin@arriam.ru (P.G.K.); a.gorshkov@arriam.ru (A.P.G.); avtsyganova@arriam.ru (A.V.T.)

*Author for correspondence:

Viktor E. Tsyganov

Tel: +7 812 4705100

E-mail: vetsyganov@arriam.ru

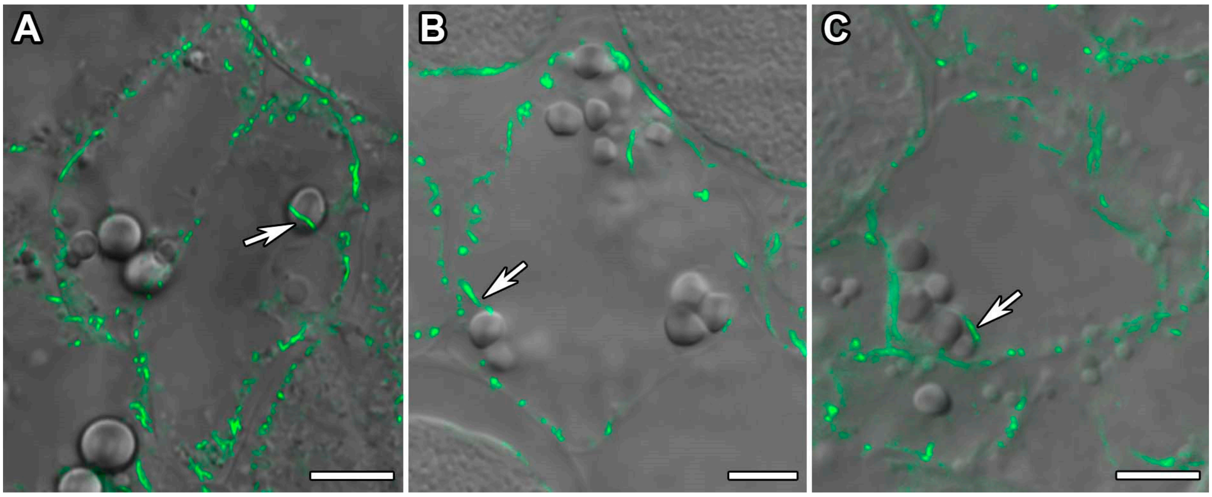

Figure S1. Endoplasmic microtubule organization in uninfected cells. (A) *Vigna radiata*, (C) *Vigna unguiculata*, (E) *Lotus corniculatus*. Confocal laser scanning microscopy of 50 μm (A, B) and 35 μm (C) longitudinal vibratome sections. Immunolocalization of tubulin (microtubules), green channel. Merged images of a single optical section of differential interference contrast and green channel. Arrows indicate endoplasmic microtubules. Bars are 5 μm.

Table S1. Protocol for sample preparation.

Step	Reagent	Duration
1,2,3	phosphate buffer	45 min (15 min each)
4	OsO_4	2 h
5,6,7	phosphate buffer	45 min (15 min each)
8	H_2O	5 min
9	30% ethanol	20 min
10	50% ethanol	20 min
11	70% ethanol	20 min
12	90% ethanol	20 min
13	100% ethanol	20 min
14	ethanol + acetone (1:1)	10 min
15,16	100% acetone	40 min (20 min each)
17	resin + acetone (1:1)	2 h
18	resin + acetone (2:1)	2 h
19	resin + acetone (3:1)	2 h
20	100% resin	16 h

Table S2. Composition of fixative solutions.

Plant	Compounds	Time of vacuum infiltration
V. radiata	3% paraformaldehyde, 0.25% glutaraldehyde, 0.3% Tween-20, 0.3% Triton X-100, 10% DMSO in 1/10 MTSB	30 min of air pumping out, 6 min of infiltration, 7 cycles
V. unguiculata	3% paraformaldehyde, 0.25% glutaraldehyde, 0.3% Tween-20, 0.3% Triton X-100 in 1/8 MTSB	30 min of air pumping out, 6 min of infiltration, 7 cycles
L. corniculatus	3% paraformaldehyde, 0,25% glutaraldehyde, 0,3 Tween-20, 0,3% Triton X-100 in 1/10 MTSB	30 min of air pumping out, 6 min of infiltration, 5 cycles

MTSB – 50 mM PIPES, 5 mM $MgSO_4 \cdot 7H_2O$, 5 mM EGTA, pH 6.9
